# Burnout among teachers in three Canadian Provinces: Findings from the Wellness4Teachers Support Program

**DOI:** 10.1192/j.eurpsy.2025.389

**Published:** 2025-08-26

**Authors:** A. Belinda, R. D. L. Dias, Y. Wei, V. I. O. Agyapong

**Affiliations:** 1 University of Alberta, Edmonton; 2 Dalhousie University, Halifax, Canada

## Abstract

**Introduction:**

Burnout is a longstanding issue among educators and has been associated with psychological and physical health problems such as depression, and insomnia.Chronic stress has been associated with burnout, defined by three dimensions of overpowering exhaustion (emotional exhaustion), cynicism (feelings of cynicism and detachment from work), and inefficacy (a sense of ineffectiveness and lack of accomplishment) which conceptualize the individual stressful experience in a social context.

**Objectives:**

To assess the prevalence and predictors of the three dimensions of burnout (emotional exhaustion, depersonalization and lack of professional accomplishment) among elementary and high school teachers.

**Methods:**

This is a quantitative cross-sectional study with data collected via an online survey. The Maslach Burnout Inventory-Educator Survey (MBI-ES), the Brief Resilience Scale (BRS) and the Perceived Stress Scale were used, respectively, to assess burnout, resilience and stress among teachers. Data was collected between September 1^st^, 2022 and August 30^th^, 2023. SPSS (version 28, IBM Corp) was used for the data analysis.

**Results:**

Overall, 1912 educators received a link to the online survey via a text message, and 780 completed the burnout survey questions, resulting in a response rate of 41%. The prevalence of emotional exhaustion, depersonalization, and lack of professional accomplishment were 76.9%, 23.2%, and 30.8%, respectively. Participants with high-stress symptoms were 6.88 times more likely to experience emotional exhaustion (OR = 6.88; 95% CI: 3.31–14.29), 2.55 times (OR = 2.55; 95% CI: 1.65–3.93) more likely to experience depersonalization and 2.34 times (OR = 2.34; 95% CI: 1.64–3.35) more likely to experience lack of professional fulfilment. Additionally, respondents with low resilience were 3.26 times more likely to experience emotional exhaustion symptoms (OR = 3.26; 95% CI: 2.00–5.31),) than those with high resilience. Males were about 2.4 times more likely to present with depersonalization compared to female teachers, whilst those who indicated their marital status as partnered or cohabiting and those who selected “other” were 3.5 and 7.3 times respectively more likely to present with depersonalization compared with those who were single. Finally, Physical Education were 3.8 times more likely to present with depersonalization compared with English teachers.

**Conclusions:**

The current study highlights the predictive effects of low resilience and high stress on the three dimensions of burnout among teachers in Canada. Interventions aimed at addressing systemic stress and fostering resilience are needed to reduce burnout among teachers.

**Disclosure of Interest:**

None Declared

